# Hospital Admission Despite Low-Risk History, Electrocardiogram, Age, Risk factors, and Troponin (HEART) Scores and 30-Day Outcomes in Emergency Department Chest Pain

**DOI:** 10.1016/j.acepjo.2026.100502

**Published:** 2026-09-04

**Authors:** Hao Wang, Eric Chou, Richard D. Robinson, Nicholas Saltarelli, Garrett Johnson, Sarah Merchant, Emily Morales, Jessica J. Kirby

**Affiliations:** a Department of Emergency Medicine, JPS Health Network, Fort Worth, Texas, USA; b Department of Emergency Medicine, Baylor Scott & White All Saints Hospital, Fort Worth, Texas, USA; c Department of Emergency Medicine, Baylor University Medical Center, Dallas, Texas, USA

**Keywords:** chest pain, HEART score, emergency department, risk stratification, major adverse cardiovascular events

## Abstract

**Objective:**

To evaluate whether hospital admission of emergency department (ED) patients despite low-risk History, Electrocardiogram, Age, Risk factors, and Troponin (HEART) scores (<4) identifies a subgroup at higher risk for 30-day major adverse cardiovascular events (MACE) and to determine whether clinician override provides prognostic information beyond the HEART score.

**Methods:**

We conducted a retrospective, multicenter observational study across 6 EDs from January 1, 2021, through December 31, 2023. Adult patients presenting with chest pain and a documented HEART score were included. Analyses were restricted to first encounters among patients with low-risk HEART scores (<4). Clinician override was defined as hospital admission despite low-risk classification. The primary outcome was 30-day MACE, defined as myocardial infarction (MI), coronary revascularization, or all-cause mortality. Multivariable logistic regression identified factors associated with clinician override and evaluated the independent association between clinician override and 30-day MACE after adjustment for age, sex, race or ethnicity, study site, HEART score variables, troponin trend, chronic heart failure, and chronic kidney disease. Incremental prognostic value was assessed using the likelihood ratio and DeLong tests.

**Results:**

Among 33,392 patients with low-risk HEART scores, clinician override occurred in 2101 (6.3%). Thirty-day MACE occurred in 120 patients (0.36%), including 40 of 2101 admitted patients (1.90%) and 80 of 31,291 discharged patients (0.26%). Clinician override was independently associated with higher odds of 30-day MACE (adjusted odds ratio, 5.16; 95% CI, 3.40 to 7.82). Clinician override significantly improved prediction of 30-day MACE beyond the baseline clinical model (AUROC, 0.8006 vs. 0.8287; DeLong *P* = .0026; likelihood ratio χ^2^ = 49.82, *P*<.001).

**Conclusion:**

Among ED patients with low-risk HEART scores, clinician override was uncommon but identified a subgroup at substantially higher risk for 30-day MACE. Although clinician override was largely associated with measurable clinical characteristics, it provided incremental prognostic information beyond the HEART score and major comorbidities, supporting the complementary role of clinician judgment in risk stratification.

**The Bottom Line**

The History, Electrocardiogram, Age, Risk factors, and Troponin (HEART) score is widely used to identify emergency department patients with chest pain who can be safely discharged, but some patients with low-risk scores are still admitted. We studied 33,392 adults with low-risk HEART scores (<4) across 6 emergency departments. Only 6.3% were admitted despite being classified as low risk, but these patients had higher 30-day rates of major adverse cardiovascular events (1.90% vs. 0.26%). Clinician override was largely associated with higher-risk clinical features but remained independently associated with >5-fold higher odds of 30-day major adverse cardiovascular events after adjustment for baseline cardiovascular risk, suggesting that clinician judgment provides incremental prognostic information beyond the HEART score.

## 1 Introduction

### 1.1 Background

Chest pain is one of the most common reasons for emergency department (ED) evaluation and a major contributor to hospital admissions, diagnostic testing, and health care expenditures.^1^ Accurate risk stratification is essential to minimize missed short-term major adverse cardiovascular events (MACE), while avoiding unnecessary admissions among patients at low risk for acute coronary syndrome.^1^^,^^2^

The History, Electrocardiogram, Age, Risk factors, Troponin (HEART) score is widely used to guide ED risk stratification.^3^ Patients with low-risk HEART scores are often considered appropriate for early discharge and outpatient follow-up, although reported short-term MACE rates have varied across studies.^4^, ^5^, ^6^ A prior systematic review and meta-analysis of more than 11,000 patients reported a 1.6% MACE rate among patients classified as low risk, whereas studies evaluating the HEART Pathway with serial troponin testing have reported substantially lower event rates.^5^^,^^6^ In routine practice, however, clinical disposition decisions frequently extend beyond score outputs. Clinicians may admit patients despite low-risk scores when additional factors beyond HEART components are present, including evolving symptoms, dynamic electrocardiographic changes, comorbidity burden, prior records, and social context.^7^ Contemporary guidelines emphasize that risk scores should support, rather than replace, clinical judgment.^1^^,^^8^

### 1.2 Importance

Prior work comparing unaided clinician judgment with structured risk scores suggests that clinician judgment alone may be less sensitive for identifying short-term events when used to justify early discharge.^9^^,^^10^ However, in contemporary ED practice, clinicians typically apply judgment alongside structured tools and high-sensitivity troponin testing. The clinically relevant question, therefore, is not whether clinical judgment is superior to the HEART score, but how clinicians use HEART in practice, and whether clinicians' overrides among patients classified as low risk identify a subgroup at meaningfully higher short-term risk.

### 1.3 Goals of This Investigation

In routine clinical practice, however, we observed that a subset of patients classified as low risk by the HEART score were nevertheless admitted, raising the question of whether clinician override identifies a subgroup with higher short-term risk and distinct clinical characteristics. Accordingly, in a multicenter ED cohort, we evaluated outcomes among patients with low-risk HEART scores (<4). We examined whether hospital admission despite low-risk classification was associated with a higher risk of 30-day adverse cardiovascular events and whether these associations persisted after accounting for individual HEART components and troponin dynamics. By focusing on variation in clinician disposition within the low-risk HEART group, this study seeks to understand better how structured risk tools and clinician discretion interact in ED chest pain evaluation.

## 2 Methods

### 2.1 Study Design and Setting

We conducted a retrospective, multicenter observational study across 6 EDs within a regional health system between January 1, 2021, and December 31, 2023. The participating EDs included 2 tertiary referral centers and 4 community hospitals, with annual ED volumes ranging from approximately 60,000 to 130,000 visits. All sites used a unified electronic health record (EHR) platform across ED, inpatient, and outpatient settings. The study was approved by the Institutional Review Boards of the University of North Texas Health (IRB #1541042) and Baylor Scott & White Health (IRB #023061) with a waiver of informed consent. This study was conducted and reported in accordance with the Strengthening the Reporting of Observational Studies in Epidemiology (STROBE) guidelines.^11^

### 2.2 Study Population

We included adult patients (≥18 years) presenting to a participating ED with a chief complaint of chest pain or chest pain equivalent who had a History, ECG (electrocardiogram), Age, and Risk factors (HEAR) score documented during the index ED encounter as part of routine clinical care. To evaluate clinician decision-making among patients considered eligible for discharge, primary analyses were restricted to patients with low-risk HEART scores (<4). To avoid clustering by repeated visits, only the first qualifying ED encounter per patient during the study period was included. Patients were excluded if they left without being seen, left against medical advice, were transferred directly to another facility, underwent emergent cardiac catheterization during the ED visit, or died in the ED. Patients without documented HEAR scores were excluded from the analytic cohort. In addition, to reduce potential confounding from clearly identifiable noncardiac causes of chest pain, we excluded patients whose admission or discharge diagnoses indicated alternative etiologies, including traumatic conditions (e.g., rib fracture, pneumothorax), pneumonia, pulmonary embolism, aortic dissection, pericarditis, costochondritis, etc., based on International Classification of Diseases, 10th Revision (ICD-10) diagnosis codes.

### 2.3 HEART Score Assessment

HEAR scores were calculated prospectively by treating clinicians and documented in the EHR prior to disposition. The HEAR score includes 4 components: history, electrocardiogram (ECG), age, and risk factors. The HEAR score is embedded as a structured tool within the EHR and is used as a quality metric across the participating EDs. Clinicians are encouraged to complete and document the HEAR score when evaluating patients with suspected cardiac chest pain. Documented HEAR scores and their individual components were electronically retrieved from structured EHR fields. The HEART scores used in this study were those prospectively entered by treating clinicians during routine clinical care, and no retrospective recalculation or independent verification was performed. High-sensitivity cardiac troponin assays were used at all sites. Five EDs used a high-sensitivity cardiac troponin I (hs-cTnI) assay with sex-specific 99th percentile upper reference limits of 78 ng/L for males and 53 ng/L for females, whereas one ED used a high-sensitivity cardiac troponin T (hs-cTnT) assay with a 99th percentile upper reference limit of 15 ng/L for both sexes. Troponin values were interpreted relative to the assay- and sex-specific 99th percentile upper reference limit applicable at each site. Troponin scores were categorized as 0 (below the 99th percentile), 1 (1 to <3 times the 99th percentile), or 2 (≥3 times the 99th percentile). The primary troponin scoring approach was based on prior work comparing modified HEART score approaches using high-sensitivity troponin, which found that this method identified the largest number of low-risk chest pain patients, while maintaining similar diagnostic accuracy for MACE prediction.^12^ As a sensitivity analysis, we recalculated HEART scores using a binary high-sensitivity troponin scoring system, assigning 0 points to troponin values below the assay-specific 99th percentile upper reference limit and 2 points to positive troponin values. Patients could have ≤3 high-sensitivity troponin measurements obtained during the ED encounter, depending on the duration of the ED stay and clinical assessment. If multiple troponin values were measured during the index ED visit, the HEART score was calculated based on the last ED troponin value. Troponin trend change was defined as a rise or fall of ≥ 15 ng/L between serial measurements during the ED encounter. For the analysis examining factors associated with clinician override, each HEART score component was entered into the multivariable model using its original scoring (0, 1, or 2) to preserve the ordinal information in the HEART score. For analysis evaluating 30-day MACE, the overall HEART score was included as a continuous covariate representing baseline cardiovascular risk.

### 2.4 Definition of Clinician Override

Clinician disposition decisions were determined by the treating physician and were not protocolized based on HEART score results. Clinician override was defined as hospital admission despite a low-risk HEART score (<4). Patients with HEART scores <4 who were discharged from the ED served as the reference group.

### 2.5 Outcome

The primary outcome was 30-day MACE, defined as a composite of acute myocardial infarction (MI), coronary revascularization (percutaneous coronary intervention [PCI] or coronary artery bypass grafting [CABG]), or all-cause mortality occurring within 30 days of the index ED visit. As a secondary outcome, we evaluated a narrower composite of 30-day death or MI, excluding coronary revascularization because revascularization may be influenced by subsequent diagnostic testing and treatment pathways that differ between admitted and discharged patients. Owing to the limited number of death or MI events, no adjusted multivariable analysis was performed for this secondary outcome. Thirty-day outcomes were ascertained using available records within the health system EHR and records accessible through the EHR’s Care Everywhere function from participating external health systems.

### 2.6 Covariates

Covariates were selected a priori based on clinical relevance and included age, modeled as a continuous variable to preserve information and avoid unnecessary categorization; sex (male or female), race and ethnicity (non-Hispanic White, non-Hispanic Black, Hispanic/Latino, or other), individual HEART components (history, ECG, risk factors, troponin), troponin trend (dynamic vs no dynamic change), and presence of chronic heart failure and kidney disease. Congestive heart failure (CHF) and chronic kidney disease (CKD) were identified using ICD-10 diagnosis codes recorded in the EHR. New-onset heart failure was defined as a heart failure diagnosis during the index encounter without a documented prior history of CHF in the available EHRs. CHF and CKD were analyzed as separate covariates because of their distinct clinical implications and potential independent associations with clinician decision-making and patient outcomes.

### 2.7 Statistical Analysis

Baseline characteristics and outcomes were compared between clinician-override and no-override groups using between-group differences with corresponding 95% CI. For categorical variables, absolute percentage-point differences and 95% CIs were calculated. For continuous variables, between-group differences and corresponding 95% confidence intervals were reported. For the overall HEART score, the between-group median difference and 95% CI were estimated using median regression with bootstrap resampling (1000 replications).

Multivariable logistic regression analyses were performed for 2 prespecified objectives. First, clinician override (hospital admission despite a low-risk HEART score) was modeled as the dependent variable to identify clinical factors independently associated with clinicians' admission decisions. Candidate variables were selected a priori based on clinical relevance and included demographic characteristics, individual HEART score components, troponin trend patterns, CHF, CKD, and study site. No automated variable selection procedures (e.g., forward, backward, or stepwise selection) were used, and no statistical significance threshold was applied for variable retention. Instead, all prespecified clinically relevant variables were entered simultaneously into the multivariable model. Model discrimination was assessed using the area under the receiver operating characteristic curve (AUROC), and calibration was evaluated using the Hosmer–Lemeshow goodness-of-fit test.

Second, multivariable logistic regression was used to evaluate whether clinician override was independently associated with 30-day MACE after adjustment for baseline clinical risk. This model included clinician override, age (continuous), sex, race or ethnicity, study site, overall HEART score, CHF, and CKD. Incremental prognostic value of clinician override was assessed by comparing model discrimination (AUROC) between models with and without clinician override using DeLong's test for correlated ROC curves. Improvement in overall model fit was evaluated using the likelihood ratio test. Adjusted odds ratios (aORs) with corresponding 95% CI were reported.

There were no missing values among the variables included in the multivariable models. Patients with unavailable or undocumented race or ethnicity information were retained as an “Unknown” category. Therefore, no imputation was performed. All analyses were conducted using Stata version 14.2 (StataCorp).

## 3 Results

### 3.1 Study Population

During the study period, 138,956 ED chest pain encounters with high-sensitivity cardiac troponin testing were identified. Of these, 57,906 encounters had a documented HEART score and met the diagnosis-based screening criteria. After exclusion of 10,025 repeated encounters, 47,881 unique patients remained. Among these, 33,392 patients (69.7%) had low-risk HEART scores (<4) and comprised the analytic cohort (Fig). Within the low-risk cohort, 2101 patients (6.3%) were admitted despite low-risk classification (clinician override), whereas 31,291 (93.7%) were discharged from the ED. Thirty-day MACE occurred in 120 of 33,392 low-risk patients (0.36%). Thirty-day death or MI occurred in 42 patients (0.13%) and is reported as a secondary descriptive outcome.

**Figure:**
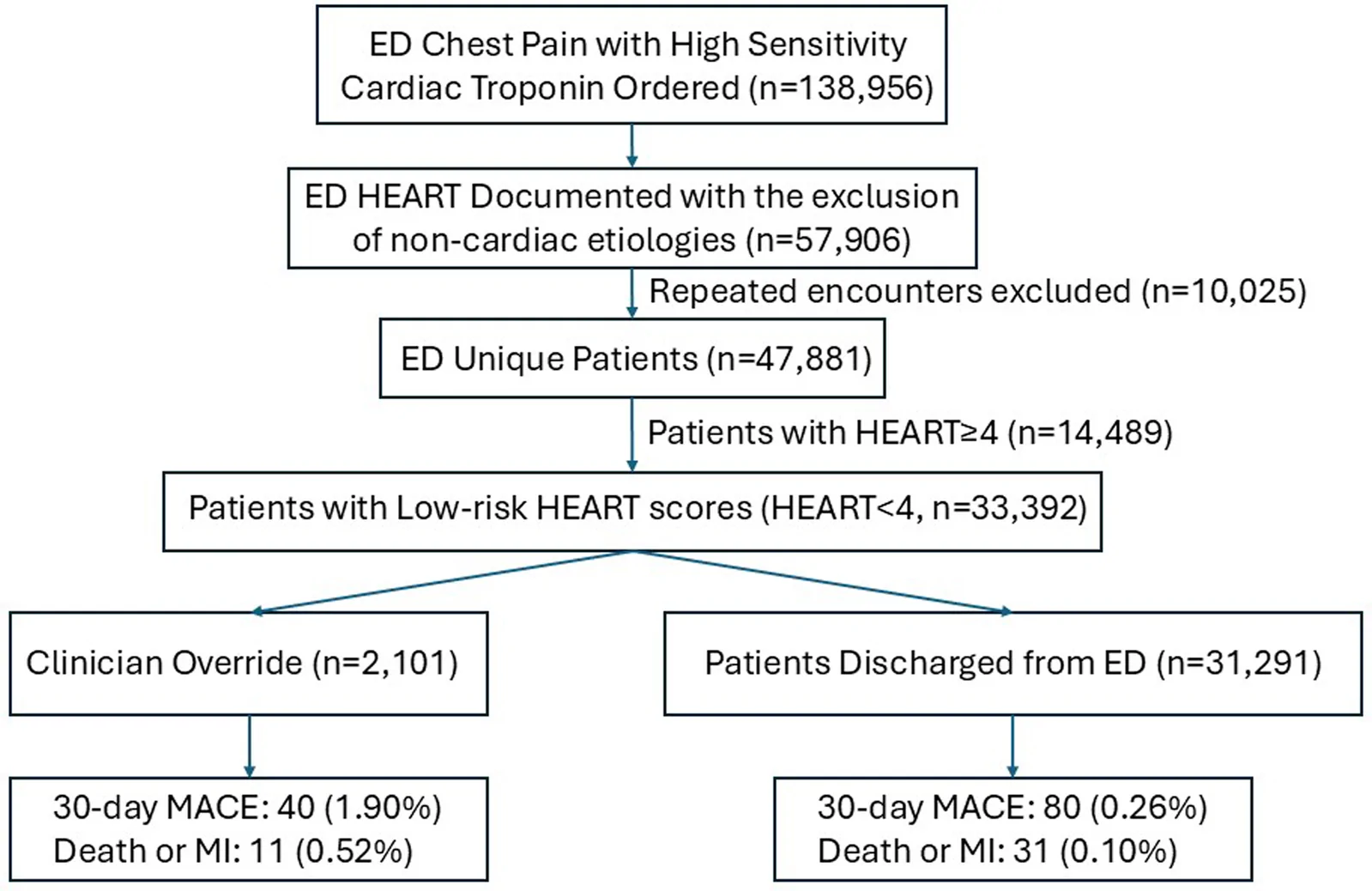
Flow of patients through the study cohort.

### 3.2 Baseline Characteristics by Disposition

Baseline characteristics stratified by disposition are shown in Table 1. Patients admitted despite low-risk HEART scores were slightly older (mean 49 vs 45 years) and more likely to be male (45.7% vs 43.3%). Racial and ethnic distributions differed modestly between groups. The overall HEART score was also higher among patients in the clinician-override group than among those in the no-override group (median [IQR], 3 [2–3] vs. 2 [1–2]); point estimate difference 1 (0.86–1.14). They were also more likely to exhibit higher-risk individual HEART components, including high-suspicion histories, abnormal ECG findings, elevated troponin levels, and greater comorbidity burden (Table 1). Additionally, dynamic troponin changes and pre-existing CHF or CKD were more common among admitted patients (Table 1). Overall, these findings indicate that clinicians preferentially admitted patients with high-risk clinical features despite an overall low-risk HEART score.

**Table 1:** Baseline characteristics of patients with low-risk HEART scores by disposition.

Characteristics	Override (n = 2101)	No override (n = 31,291)	∗Absolute difference (95% CI)
Age, (y)			
Mean (SD)	49 (14)	45 (14)	4.0 (3.4–4.6)
Sex, n (%)			
Male	961 (45.7)	13,537 (43.3)	2.5 (0.3–4.7)
Female	1140 (54.3)	17,754 (56.7)	−2.5 (−4.7 to −0.3)
Race and ethnicity, n (%)			
Non-Hispanic White	827 (39.4)	10,157 (32.5)	6.9 (4.8 to 9.1)
Non-Hispanic Black	618 (29.4)	9673 (30.9)	−1.5 (−3.5 to 0.5)
Hispanic/Latino	541 (25.8)	9847 (31.5)	−5.7 (−7.7 to−3.8)
Others	115 (5.5)	1,614 (5.2)	0.3 (−0.7 to 1.3)
HEART score – median (IQR)	3 [2–3]	2 [1–2]	1 (0.86–1.14)
HEART Component, n (%)			
History (nonlow risk suspicious, H-score =1 or 2)	632 (29.7)	3420 (10.9)	19.2 (17.2–21.1)
ECG (abnormal, E-score =1 or 2)	759 (36.1)	6229 (19.9)	16.2 (14.1–18.3)
Risk Factors (yes, R-score =1 or 2)	1634 (77.8)	19,932 (63.7)	14.1 (12.2–15.9)
Troponin (positive, T-score =1 or 2)	121 (5.8)	90 (0.3)	5.5 (4.5–6.5)
Troponin dynamic changes (Yes), n (%)	102 (4.9)	33 (0.1)	4.8 (3.8–5.7)
CHF (Yes), n (%)	134 (6.4)	517 (1.7)	4.7 (3.7–5.8)
CKD (Yes), n (%)	169 (8.0)	682 (2.2)	5.9 (4.7–7.0)

∗ for continuous variables, between-group differences were reported as point estimates with 95% CIs, including the mean difference for age and the median difference for HEART score. For categorical variables, between-group differences were reported as percentage-point differences with 95% CIs.

### 3.3 Thirty-Day Outcomes by Disposition

Thirty-day outcomes differed substantially by disposition (Table 2). Patients admitted despite low-risk HEART scores experienced higher rates of all-cause mortality (10, 0.48), coronary revascularization, and overall MACE (40, 1.90) compared with patients discharged from the ED. In contrast, MI alone was uncommon in both groups and did not differ significantly. The secondary composite outcome of death or MI also occurred more frequently among admitted patients; however, because of the limited number of events, this outcome is presented descriptively.

**Table 2:** Thirty-day MACE event rates by clinician override status.

Outcomes	Override n (%)	No-override n (%)	Percentage point difference (95% CI)
All-cause mortality	10 (0.48)	20 (0.06)	0.41 (0.12 – 0.71)
AMI	1 (0.05)	11 (0.04)	0.01 (-0.08 – 0.11)
PCI	24 (1.14)	43 (0.14)	1.00 (0.55 – 1.46)
CABG	5 (0.24)	6 (0.02)	0.22 (0.01 – 0.43)
30-day MACE	40 (1.90)	80 (0.26)	1.65 (1.06 – 2.24)

Abbreviations: AMI, acute myocardial infarction; CABG, coronary artery bypass grafting; CI, confidence interval; MACE, major adverse cardiovascular events; PCI, percutaneous coronary intervention.

In a sensitivity analysis using binary high-sensitivity troponin scoring, 33,244 patients were classified as low risk, compared with 33,392 patients in the primary analysis. Thirty-day MACE occurred in 1.89% of patients in the clinician-override group compared with 0.25% in the no-override group, corresponding to an absolute difference of 1.63 percentage points (95% CI, 1.04 to 2.23). These findings were consistent with the primary analysis (Supplemental Table S1).

### 3.4 Factors Associated with Clinician Override

To better understand the clinical factors associated with clinician override, we developed a multivariable logistic regression model with clinician override as the dependent variable (Table 3). Increasing HEART score component values for history, ECG, risk factors, and troponin were each independently associated with a greater likelihood of hospital admission despite an overall low-risk HEART score. Among the individual HEART score components, troponin demonstrated the strongest association with clinician override. Compared with patients with a troponin score of 0, those with a troponin score of 2 had markedly higher odds of override (adjusted OR, 88.23; 95% CI, 15.79 to 492.97). This large effect size reflects the fact that 19 of the 21 patients (90.5%) with a troponin score of 2 were admitted despite an overall low-risk HEART score. Dynamic troponin changes and the presence of CHF and CKD were also independently associated with clinician override. The model demonstrated good discrimination (AUROC 0.839) and satisfactory calibration (Hosmer–Lemeshow *P*=.505), indicating that clinicians' admission decisions were primarily explained by measurable clinical characteristics.

**Table 3:** Multivariable model of factors associated with clinician override.

Variables	Adjusted odds ratio (95% CI)
Model outcome	Clinician override
Age (from HEART score)	
1 vs. 0	1.14 (1.02–1.27)
2 vs. 0	1.43 (1.17–1.74)
Female sex	1.01 (0.92–1.12)
Race or ethnicity	
NHB vs NHW	0.75 (0.66–0.85)
Hispanic vs NHW	0.70 (0.61–0.79)
Other vs NHW	0.89 (0.71–1.11)
History (from HEART score)	
1 vs. 0	2.63 (2.33–2.96)
2 vs. 0	11.76 (7.48–18.49)
ECG (from HEART score)	
1 vs. 0	2.28 (2.04–2.55)
2 vs. 0	9.34 (4.42–19.74)
Risk factors (from HEART score)	
1 vs. 0	1.51 (1.33–1.71)
2 vs. 0	2.48 (2.05–3.00)
Troponin (from HEART score)	
1 vs. 0	7.74 (5.37–11.15)
2 vs. 0	88.23 (15.79–492.97)
Troponin pattern	
Only a single troponin tested at ED	Reference
Serial troponin tested with no changes at ED	6.42 (5.75–7.17)
Serial troponin tested with dynamic changes at ED	62.17 (39.21-98.58)
Comorbidities	
CHF only vs. neither	2.09 (1.59–2.73)
CKD only vs. neither	2.27 (1.79–2.87)
Both vs. neither	2.81 (1.86–4.25)
ED locations	
Site 2 vs. Site1	0.43 (0.37–0.51)
Site 3 vs. Site 1	0.31 (0.26–0.37)
Site 4 vs. Site 1	0.27 (0.23–0.32)
Site 5 vs. Site 1	0.20 (0.16–0.24)
Site 6 vs. Site 1	1.05 (0.90–1.22)

Abbreviations: CI, confidence interval; MI, myocardial infarction; MACE, major adverse cardiac event; NHB, Non-Hispanic Black; NHW, Non-Hispanic White; ECG, electrocardiogram; CHF, congestive heart failure; CKD, chronic kidney disease.

### 3.5 Association between clinician override and 30-Day MACE

After adjustment for age, sex, race or ethnicity, study site, overall HEART score, CHF, and CKD, clinician override remained independently associated with increased odds of 30-day MACE (adjusted OR 5.16; 95% CI 3.40 to 7.82; Table 4). Higher HEART score, CHF, and CKD were also independently associated with increased MACE risk.

**Table 4:** Multivariable logistic regression of clinician override and 30-Day MACE among ED patients with low-risk HEART scores.

Variables	Adjusted odds ratio (95% CI)
Model outcome	30 day-MACE
Clinician override (admitted vs. discharged)	5.16 (3.40–7.82)
Age (per year)	1.02 (1.01–1.04)
Female sex	1.53 (1.06–2.20)
Race or ethnicity	
NHB vs. NHW	0.45 (0.27–0.75)
Hispanic vs. NHW	0.67 (0.41–1.08)
Other vs. NHW	0.99 (0.47–2.10)
HEART score	1.68 (1.28–2.21)
ED Location	
Site 2 vs. Site 1	3.13 (1.17–8.34)
Site 3 vs. Site 1	3.55 (1.32–9.58)
Site 4 vs. Site 1	4.49 (1.71–11.80)
Site 5 vs. Site 1	3.45 (1.26–9.39)
Site 6 vs. Site 1	3.05 (1.15–8.09)
CHF(positive vs. negative)	2.43 (1.31–4.50)
CKD (positive vs. negative)	1.90 (1.01–3.56)

Abbreviations: CI, confidence interval; MI, myocardial infarction; MACE, major adverse cardiac event; NHB, non-Hispanic Black; NHW, non-Hispanic White; CHF, congestive heart failure; CKD, chronic kidney disease.

Adding clinician override to the baseline clinical model improved discrimination for 30-day MACE (AUROC 0.8006 vs. 0.8287; ΔAUROC 0.0281; DeLong *P* =.0026). Model fit also improved significantly (likelihood ratio χ^2^=49.82, *P*<.001), demonstrating that clinician override provided incremental prognostic information beyond the HEART score and major comorbidities.

## 4 Limitations

Several limitations merit consideration. First, as a retrospective observational study, residual confounding is possible. Although patients with identifiable alternative causes of chest pain were excluded using admission and discharge diagnosis codes, diagnosis-code-based screening may not capture all alternative etiologies or the specific clinical reason for admission, including persistent or recurrent symptoms, changes in symptoms during the ED stay, subtle ECG findings not represented by the HEART ECG component, hemodynamic concerns, prior cardiac testing or known coronary anatomy, clinician gestalt, and concerns regarding the feasibility of timely outpatient follow-up. Therefore, residual confounding from unmeasured diagnoses and other clinical factors influencing disposition remains possible. HEART scores were based on clinician-entered values and were not independently recalculated or verified; therefore, interclinician variability, particularly in subjective components, such as the History component, and potential scoring errors cannot be excluded. Furthermore, although our additional multivariable analysis demonstrated that clinician override was largely explained by measurable clinical characteristics, including HEART score components, troponin dynamics, and major comorbidities, clinician decision-making may still reflect unmeasured factors that were unavailable in the EHR, such as symptom evolution during the ED stay, clinician assessment of patient reliability, frailty, prior cardiac testing, social circumstances, or anticipated barriers to timely outpatient follow-up. Therefore, residual confounding from these unmeasured factors cannot be excluded.

Second, the composite MACE outcome included coronary revascularization, which may be influenced by admission decisions and subsequent diagnostic testing. Therefore, some observed differences in MACE may partially reflect differences in downstream clinical management rather than underlying disease severity alone. In addition, all-cause mortality was included in the MACE and death-or-MI composite outcomes, because reliable adjudication of cause-specific mortality was not available within the EHR. Consequently, some deaths may have been unrelated to acute coronary syndrome or cardiovascular disease.

Third, although outcomes were ascertained using records within the health system EHR and available external records through Care Everywhere, events or deaths occurring outside the health system and unavailable through connected external records may not have been captured. Importantly, incomplete outcome ascertainment may have differed according to index disposition. Admitted patients had a greater opportunity for events to be identified during hospitalization and subsequent care within the health system, whereas discharged patients may have experienced events at outside facilities or deaths outside the hospital that were not captured in the available records. Such differential misclassification of outcomes could bias the observed association between clinician override and 30-day outcomes. Given the low absolute number of events, even a small number of missed outcomes could influence the magnitude and precision of the observed associations, and the direction and extent of this potential bias could not be quantified.

Fourth, inclusion was limited to encounters with documented HEART scores, and HEART score documentation may not have been uniform across all ED chest pain presentations. Although HEART score documentation was encouraged as a quality metric, the decision to complete the score remained at the treating clinician's discretion. Therefore, patients perceived as having a higher likelihood of acute coronary syndrome or those considered most appropriate for HEART-based risk stratification may have been more likely to have a documented score, introducing potential selection bias and limiting generalizability to all ED patients with chest pain. Finally, although the study included diverse ED settings, all sites were within a single health system, which may limit generalizability. External validation in independent health systems is warranted.

## 5 Discussion

In this multicenter cohort of ED patients with low-risk HEART scores, clinicians admitted only a small proportion of patients (6.3%) despite low-risk classification. Clinician override was strongly associated with measurable clinical characteristics, including higher-risk HEART score components, dynamic troponin changes, and the presence of CHF and CKD. Nevertheless, after adjustment for baseline cardiovascular risk, as represented by the overall HEART score, and major comorbidities, clinician override remained independently associated with >5-fold higher odds of 30-day MACE and significantly improved model discrimination. These findings suggest that clinician disposition decisions were largely based on identifiable clinical features and captured additional prognostic information beyond structured risk assessment alone.

Although patients with identifiable alternative causes of chest pain were excluded based on admission and discharge diagnosis codes, the observed association should not be interpreted as indicating that clinicians override specifically identified occult acute coronary syndrome. Rather, admission decisions may reflect broader clinical risk signals and patient-specific factors not fully captured by the HEART score or the measured covariates.

Our findings provide insight into how clinicians apply the HEART score in routine practice. Rather than overriding the HEART recommendation arbitrarily, clinicians preferentially admitted patients with higher-risk clinical characteristics despite an overall HEART score of <4. The multivariable override model demonstrated that increasing HEART component scores, dynamic troponin changes, CHF, and CKD were independently associated with admission decisions, indicating that clinicians systematically incorporated these measurable risk signals with additional contextual factors, such as symptom evolution, prior records, subtle ECG changes, or overall clinical gestalt, that are not easily quantified within structured scoring systems when deviating from the recommended low-risk disposition.^2^^,^^13^

This study was not designed to determine whether clinician judgment outperforms the HEART score. Rather, it evaluates how clinicians use HEART scoring in practice. Because HEART scoring informs clinical decision-making, direct comparison of score performance versus clinician disposition would be subject to incorporation bias.^14^ By restricting analysis to patients with low-risk scores and examining variation in clinician disposition within this group, we sought to evaluate how clinicians modify risk assessment beyond the structured tool. Our findings support a complementary model, in which structured scores guide initial stratification while allowing clinician discretion to incorporate additional patient-specific information.

The absolute event rates in this cohort were low, consistent with prior HEART validation studies demonstrating favorable short-term outcomes among patients with low-risk.^6^^,^^15^ Thus, the overall safety of HEART-based discharge strategies is reaffirmed. However, our analysis addressed a different question by examining variation in outcomes across clinician dispositions within the low-risk group. Differences across prior studies may also reflect variation in patient selection, troponin assays and scoring approaches, serial testing protocols, and outcome definitions.^5^^,^^6^ At the same time, the small subset of patients selected for admission had a higher observed risk, suggesting that selective override may identify a clinically meaningful subgroup without broadly undermining the efficiency of HEART-guided pathways.^16^^,^^17^

## 6 Conclusion

In summary, among ED patients with low-risk HEART scores, clinician override was strongly associated with measurable clinical risk factors and remained independently associated with 30-day MACE after adjustment for baseline cardiovascular risk. These findings suggest that clinician judgment complements structured HEART-based risk assessment by incorporating additional prognostic information beyond the composite score alone. Future prospective studies are needed to identify the factors underlying clinician override and determine whether incorporating them into existing risk assessment tools can further improve clinical decision-making.

## Author Contributions

HW: Conceptualization, Data curation, Formal analysis, Investigation, Methodology, Project administration, Validation, Visualization, Writing-original draft, and Writing-review & editing. EC: Conceptualization, Data curation, Investigation, Methodology, Project administration, Validation, and Writing-review & editing. RDR: Conceptualization, Data curation, Investigation, Methodology, Project administration, Validation, and Writing-review & editing. NS: Conceptualization, Project administration, Validation, and Writing-review & editing. GJ: Conceptualization, Project administration, Validation, and Writing-review & editing. SM: Investigation, Project administration, and Writing-review & editing. EM: Investigation, Project administration, and Writing-review & editing. JJK: Investigation, Project administration, and Writing-review & editing.

## Funding and Support

By *JACEP Open* policy, all authors are required to disclose any and all commercial, financial, and other relationships in any way related to the subject of this article as per ICMJE conflict of interest guidelines (see www.icmje.org). The authors have stated that no such relationships exist.

## Conflicts of Interest

All authors have affirmed they have no conflicts of interest to declare.
