# Supplementary material for: Hospital Admission Despite Low-Risk History, Electrocardiogram, Age, Risk factors, and Troponin (HEART) Scores and 30-Day Outcomes in Emergency Department Chest Pain
Source: J Am Coll Emerg Physicians Open. 2026 Sep 4;7(5):100502. doi: 10.1016/j.acepjo.2026.100502 (PMC13571236; doi:10.1016/j.acepjo.2026.100502)
Supplement: Supplementary file 1 [file mmc1.docx]

Supplemental Table S1. Different MACE Event Rates by Overrides

| Outcomes | Override n (%) | No-Override n (%) | Percentage point difference (95% CI) |
| --- | --- | --- | --- |
| All-cause Mortality | 10 (0.50) | 20 (0.06) | 0.43 (0.12 - 0.74) |
| AMI | 1 (0.05) | 10 (0.03) | 0.02 (-0.08 – 0.12) |
| PCI | 22 (1.09) | 42 (0.13) | 0.96 (0.50 – 1.41) |
| CABG | 5 (0.25) | 6 (0.02) | 0.23 (0.01 – 0.45) |
| 30-day MACE | 38 (1.89) | 78 (0.25) | 1.63 (1.04 – 2.23) |

Footnote: AMI, acute myocardial infarction; CABG, coronary artery bypass grafting; CI, confidence interval; MACE, major adverse cardiovascular events; PCI, percutaneous coronary intervention.
